# Circulating T-Cell Receptor Excision Circles at Birth and Risk of Childhood Cancers

**DOI:** 10.3390/cancers17172903

**Published:** 2025-09-04

**Authors:** Jun Tao, Paul S. Albert, Nellie Gottlieb, Paige Miller, Eric A. Engels

**Affiliations:** 1 Division of Cancer Epidemiology and Genetics, National Cancer Institute, National Institutes of Health, Rockville, MD 20850, USA; albertp@mail.nih.gov (P.S.A.); engelse@exchange.nih.gov (E.A.E.); 2 California Cancer Reporting and Epidemiologic Surveillance (CalCARES) Program, University of California Davis Comprehensive Cancer Center, Sacramento, CA 95838, USA; ngottlieb@health.ucdavis.edu; 3 Cancer Epidemiology and Surveillance Branch, Texas Department of State Health Services, Austin, TX 78735, USA; paige.miller@dshs.texas.gov

**Keywords:** T-cell receptor excision circle, childhood cancer, pediatric cancer, newborn screening, immunodeficiency

## Abstract

Background: T-cell receptor excision circles (TRECs) are measured in newborn screening programs in the United States to identify severe combined immunodeficiency (SCID). We hypothesized that relatively low TREC levels at birth, even within the normal range, could indicate compromised immunity and higher susceptibility to childhood cancers. Methods: We conducted a case–control study using linked data from the newborn screening programs and cancer registries in California and Texas to examine the association between TREC levels and risk of childhood cancer. The study included 2196 cancer cases and 10,980 controls from California and 1186 cancer cases and 5890 controls from Texas. Results: In California, acute myeloid leukemia cases had significantly lower TREC levels compared with their matched controls (*p* = 0.0051), while in Texas, acute lymphocytic leukemia cases had significantly higher TREC levels compared with their matched controls (*p* = 0.0034). However, each association was not replicated in the other state, and other cancer types did not show significant differences in TREC levels between cases and controls. Conclusions: We did not observe consistent associations between TREC levels at birth and childhood cancer risk. A possible explanation for the lack of more clear-cut differences in TREC levels between cases and matched controls might be the complex etiology of childhood cancers. The results underscore the need for longitudinal studies that incorporate additional immune biomarkers to understand the immunologic basis of childhood cancer development.

## Simple Summary

Circulating T-cell receptor excision circles (TRECs), which can serve as a biomarker of thymic output and T-cell production, are routinely measured in newborn screening programs in the United States to detect severe combined immunodeficiency. To examine whether variation in T-cell production at birth is associated with childhood cancer risk, we conducted a population-based case–control study linking newborn TREC measurements from California and Texas with state cancer registries. In California, children who later developed acute myeloid leukemia had significantly lower TREC levels than matched controls (*p* = 0.0051), whereas in Texas, children with acute lymphoblastic leukemia had significantly higher TREC levels than controls (*p* = 0.0034). Neither association was replicated in the other state, and TREC levels were not associated with the risk of six other common cancer types in either state. These negative findings underscore the need for longitudinal studies integrating additional immune biomarkers to elucidate cancer etiology among children.

## 1. Introduction

Acquired immunosuppression is associated with a higher risk of multiple types of cancer, mainly due to impaired T-cell-mediated immune surveillance of oncogenic viruses [1]. This pattern of cancer risk is well documented in observational studies of people with acquired immunosuppressed status, including acquired immunodeficiency syndrome (AIDS, due to infection with human immunodeficiency virus) and solid organ transplantation (where recipients must be prescribed immunosuppressant medications to prevent graft rejection) [1]. Children with AIDS have an elevated risk of Kaposi sarcoma (caused by Kaposi sarcoma herpesvirus), non-Hodgkin lymphoma, and leiomyosarcoma (both of which are caused by Epstein–Barr virus) [2]. Similarly, pediatric solid organ transplant recipients have a markedly increased risk of non-Hodgkin lymphoma [3]. 

Primary immunodeficiency disorders (PIDs), which are rare inherited disorders, are also associated with elevated cancer risk [4,5,6,7]. PIDs are a heterogeneous group of diseases, including, for example, severe combined immunodeficiency (SCID) and Bloom syndrome, that increase susceptibility to recurrent infections, allergies, and autoimmunity [4,5]. Children with PIDs have a lifetime cancer risk of 5–25%, particularly for lymphomas and leukemias [4,5,7]. To date, however, no studies have prospectively investigated whether normal variation in immunity present in early infancy is associated with subsequent cancer risk among children.

Circulating T-cell receptor excision circles (TRECs) are DNA fragments generated during T-cell maturation in the thymus [8]. TRECs are not duplicated with T-cell division, so their quantification is a reflection of the number of recent thymic emigrant T-cells, and TRECs can thus be used as a biomarker of thymic function and T-cell production [8]. Extremely low TREC levels at birth indicate compromised thymic output due to SCID, and TREC testing has been widely implemented in newborn screening programs for SCID across the United States [9].

Of interest, adults who have undergone thymectomy, while not exhibiting overt immunosuppression, nonetheless have lower TREC levels, a reduced diversity of T-cell repertoire, and higher susceptibility to developing cancer [10]. We therefore hypothesized that low TREC levels at birth, while not so low as to indicate the presence of SCID, would nonetheless be a marker of decreased thymic function and might be associated with elevated cancer incidence during early childhood. To test this hypothesis, we conducted a case–control study linking newborn screening data with cancer registry records in two large states in the United States (California and Texas).

## 2. Materials and Methods

We linked cancer cases identified in the state cancer registries to the state newborn screening programs. Thus, children were included if they had TREC levels tested at birth and had sufficient identifying information in the newborn screening program database to allow linkage. All investigators were blinded to TREC levels when conducting the data linkage. The study was approved by the human subject committees at the participating state health departments.

The California Cancer Registry identified cancers among 0–5-year-old children born during 2013–2017 and followed through 2021. The Texas Cancer Registry identified cancers among 0–5-year-old children born during 2013–2018 and followed through 2018. Controls were selected from the states’ population of newborns at random and matched individually to cases in a ratio of 5:1. In California, controls were matched according to birth year, sex, and the mother’s reported race/ethnicity. In Texas, controls were matched according to birth year and sex.

From the newborn screening programs, we retrieved the TREC levels measured by quantitative polymerase chain reaction (PCR) assay on Guthrie card dried blood spots that had been obtained at birth [9,11]. The California newborn screening program first used the assay initially with a laboratory-developed assay at the Genetic Disease Laboratory in Richmond, California before 2015 and a real-time PCR assay developed by PerkinElmer Genetics after 2015, which included a positive control based on detection of the beta actin gene [9]. In California, children were identified as abnormal if their TREC level was at or below 18 copies/µL [12]. The Texas newborn screening program measured TREC levels using a laboratory-developed real-time PCR assay multiplexed with a quantitative assay for the RNase P gene [13]. The threshold at or below which children were considered screen positive for SCID was 150 copies/µL for children with birthweights above 2000 grams and 110 copies/µL for those with lower birthweights [9].

Differences in TREC levels between cases and controls were evaluated using the paired Wilcoxon signed-rank test. We analyzed data only for the eight most common cancer types, based on the combined total in California and Texas, to have a sufficient number of cases for reliable comparisons: acute lymphoblastic leukemia (ALL, N = 782 in California, N = 301 in Texas), acute myeloid leukemia (AML, N = 121 and N = 82), and cancers of the central nervous system (CNS, N = 566 and N = 359), endocrine system (N = 168 and N = 101), urinary system (N = 170 and N = 76), eye and orbit (N = 129 and N = 99), soft tissue and heart (N = 137 and N = 83), and liver and intrahepatic bile duct cancer (N = 123 and N = 85).

For cancers that showed a significant difference in TREC levels between cases and controls, we conducted additional analyses. The TREC levels at birth were categorized into tertiles (T1 [low], T2 [medium], and T3 [high]). We then used conditional logistic regression to assess associations between tertiles of TREC levels and cancer risk. To further understand the non-linear association and adjust for the variations in the absolute value of TREC levels between the two states, we calculated Z scores of the log_10_-transformed TREC levels separately for California and Texas. We then fitted generalized additive models with spline terms for the Z scores to explore the relationship between continuous TREC levels and cancer risk.

Since the Texas newborn screening program provided data from repeated TREC testing one week after birth for 6720 newborns, we also calculated the intraclass correlation coefficient (ICC) and Spearman correlation between the log_10_-transformed TREC measurements at these two time points.

Data analyses specified in advance included the paired Wilcoxon signed-rank test to compare TREC levels between cases and matched controls and the conditional logistic regression models to compare the tertiles of TREC levels. Our spline analysis to assess the possibly non-linear relationships between TREC levels and cancer risk, and the analysis of the intraclass correlation coefficient for the two-time TREC testing, were conducted post hoc. *p* < 0.05 was taken to indicate statistical significance. The statistical analyses were conducted in R version 4.2.2 (R Foundation for Statistical Computing, Vienna, Austria).

## 3. Results

We included 2196 cancer cases and 10,980 controls from California and 1186 cancer cases and 5890 controls from Texas. Cases and controls were well matched (Table 1). The mean age at cancer diagnosis was 2.6 years in California and 1.8 years in Texas. Among the children with the eight cancer types in our study, 0 cases in California and 12 cases in Texas had extremely low TREC levels at birth, indicative of a possible SCID diagnosis.

**Figure 1:**
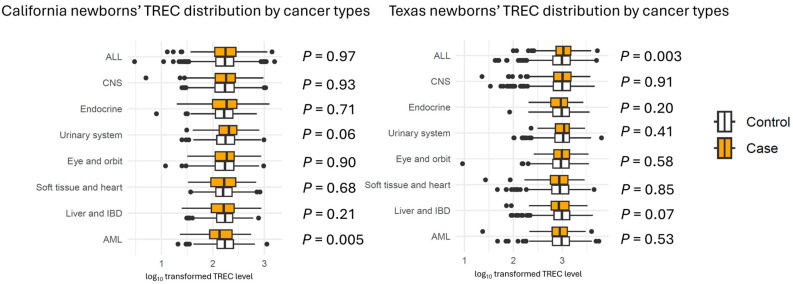
TREC levels between cases and controls for the eight most common cancer types in California and Texas. Boxplots displaying the median, 25th percentile, and 75th percentile of log_10_-transformed TREC levels among children with the eight most common types of childhood cancers and their matched controls in California and Texas. The orange bars represent cancer cases, while the white bars represent matched controls. The left panel presents data from California, and the right panel presents data from Texas. Abbreviations: ALL, acute lymphoblastic leukemia; AML, acute myeloid leukemia; CNS, central nervous system; IBD, intrahepatic bile duct.

Distributions of TREC levels overlapped substantially between cases and their matched controls for the eight most common cancer types in both California and Texas (Figure 1). In California, AML cases had significantly lower TREC levels than controls (*p* = 0.0051). In Texas, ALL cases had significantly higher TREC levels than controls (*p* = 0.0034). However, neither association was replicated in the other state, and there were no significant differences for other cancer types.

We conducted conditional regression analyses to examine the associations between tertiles of TREC levels and childhood ALL and AML (Table 2). In California, AML demonstrated an inverse association with both T2 and T3 TREC levels (odds ratio [95% confidence interval]: T2, 0.38 [0.22, 0.66]; T3, 0.47 [0.27, 0.82] vs. T1), whereas no significant association was observed in Texas. In contrast, ALL exhibited a positive association with T2 and T3 TREC levels in Texas (odds ratio [95% confidence interval]: T2, 1.45 [1.05, 1.99]; T3, 1.49 [1.06, 2.08] vs. T1), but no consistent association was found in California.

**Figure 2:**
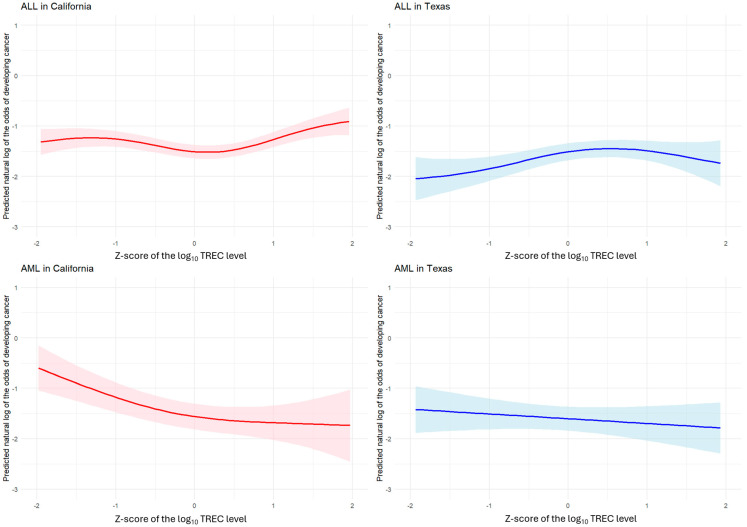
Generalized additive model plot for TREC and childhood ALL and AML in California and Texas. Plots show the continuous relationships between TREC levels and cancer risk. TREC levels were analyzed based on a Z-score of the log_10_ values (x-axis). The curves were derived using generalized additive models that incorporated spline terms for these transformed TREC levels. The y-axis corresponds to the predicted natural log of the odds of developing cancer along with the 95% confidence interval (colored band). Results are shown for AML and ALL in California (the first column) and Texas (the second column). Abbreviations: ALL, acute lymphoblastic leukemia; AML, acute myeloid leukemia.

The relationships between continuous TREC levels and the risk of childhood ALL or AML are presented using plots based on generalized additive models (Figure 2). For ALL, the trends in cancer risk were not clear-cut in either California or Texas. For AML, there appeared to be a decreasing trend in risk with higher TREC levels suggested by the plot for California, whereas the trend for Texas was essentially flat.

Based on measurements of TREC levels at birth and one week later in Texas, the ICC was 0.40, indicating moderate variation between these two time points. The Spearman correlation between the two measurements was 0.55.

## 4. Discussion

In the present case–control study, we comprehensively assessed associations between TREC levels measured at birth and subsequent cancer risk among children in California and Texas. We observed significant differences between cases and matched controls for ALL and AML in Texas and California, respectively. However, neither association showed a clear stepwise increase in risk across tertiles, and neither association was replicated in the other state. The lack of a strong “dose–response” relationship and the lack of consistency argue against a causal interpretation of our results, as pointed out by Bradford Hill [14]. Findings for the other six cancer types that we evaluated were consistently null. We presented the preliminary findings of this study at the 2025 Annual Meeting of the American Association for Cancer Research [15].

A possible explanation for the lack of stronger and more consistent differences in TREC levels between cases and matched controls might be the complex etiology of childhood cancers, encompassing not only immune status but also genetic, environmental, prenatal, and parental factors [16]. Of note, almost all children in this study had TREC levels within the normal range, indicating clinically normal thymus function, and it is possible that only more severe T-cell immunodeficiency impacts cancer risk among children. Another consideration is that the TREC levels vary somewhat over time during the newborn period, as reflected in our ICC estimate, and thymic function may vary further during subsequent early childhood.

The presence of children with Down syndrome among the population of newborns might provide an explanation for our findings for AML. Children with Down syndrome have substantially lower TREC levels at birth than otherwise normal children, and their risk of AML is greatly elevated [17,18]. Moreover, the prevalence of Down syndrome is higher in California than in Texas (16.5 vs. 13.9 per 10,000 live births) [17,18]. Thus, confounding by Down syndrome might explain the association we observed between low TREC levels and risk of AML in California and why this association was not observed in Texas. Although Down syndrome is also associated with an increased risk of ALL [17], the association between TREC levels and ALL in Texas was in the positive direction, so confounding of that association by Down syndrome is not a plausible explanation.

When TREC levels are very low, additional immunologic testing is required to document a SCID diagnosis [19]. Given that extremely low TREC levels consistent with this disorder were rare in our study population, it was not our aim to assess the associations between SCID and childhood cancers. Lymphomas account for two-thirds of malignancies among PID patients, driven by DNA repair deficits and poor control of Epstein–Barr virus infection [7]. Reintegration of TRECs might also mediate oncogenesis in immature T-cell malignancies, but such cancers are rare among children [20]. We are unaware of other studies that have directly examined associations between TREC levels and cancer incidence, although one study found that lower TREC levels were associated with worse survival among hematopoietic stem cell transplant recipients with AML [21].

Although our results are essentially negative, increasing evidence suggests that thymic function plays a critical role in cancer development [10,22,23,24]. Following adult thymectomy, patients exhibit lower TREC levels and experience a significantly higher risk for cancer and mortality when compared with patients who underwent cardiothoracic surgery without thymectomy [10]. In addition, the single nucleotide polymorphism rs2204985, a common genetic variant within the T-cell receptor locus, has been found to associate with the thymic function and TREC levels [22,25]. Furthermore, individuals with the low thymopoiesis genotype at rs2204985 (AA) had a 10% higher overall cancer risk over 10 years compared to those with the high thymopoiesis genotype (GG), including, specifically, a 14% higher risk for melanoma and a 16% higher risk for genitourinary cancers [25].

This study has important strengths, including its incorporation of prospective population-based data from two large states in the United States and comprehensive measurement of TREC levels at birth from the states’ newborn screening programs. A limitation is that there were only a small number of cases of some specific cancer types, especially those, such as lymphoma, that are caused by viruses, so we could not include them in our study. Also, we were unable to evaluate changes in TREC levels throughout early childhood, which may have revealed additional associations with cancer risk. Finally, the assays used to measure TREC levels differed in California and Texas, which could partly underlie the different results we observed for ALL and AML.

## 5. Conclusions

In summary, our study did not find consistent associations between TREC levels measured at birth and subsequent cancer risk among children. While these results are negative, they do not exclude the possibility that variation in immunity during childhood within the normal range contributes to the development of some pediatric cancers. These findings thus highlight a need to consider additional approaches, including an evaluation of longitudinal measures of immunity and the incorporation of additional immune biomarkers, to better understand the immunologic basis of childhood cancer development.

## Figures and Tables

**Table 1:** Demographic characteristics of California and Texas newborns.

	California		Texas	
	Cases, N (%)	Controls, N (%)	Cases, N (%)	Controls, N (%)
Total	2196 (100)	10,980 (100)	1186 (100)	5890 (100)
Sex				
Male	1177 (53.6%)	5885 (53.6%)	604 (50.9%)	2998 (50.9%)
Female	1019 (46.4%)	5095 (46.4%)	580 (48.9%)	2882 (48.9%)
Unknown	--	--	2 (0.2%)	10 (0.2%)
Race/ethnicity				
Non-Hispanic White	700 (31.9%)	3500 (31.9%)	497 (41.9%)	2155 (36.6%)
Non-Hispanic Black	79 (3.6%)	395 (3.6%)	93 (8.0%)	705 (12.0%)
Hispanic	1006 (45.8%)	5030 (45.8%)	462 (39.0%)	2189 (37.1%)
Asian	286 (13.0%)	1430 (13.0%)	33 (2.8%)	222 (3.8%)
Other	125 (5.7%)	125 (5.7%)	101 (8.5%)	619 (10.5%)
Birth year				
2013	504 (23.0%)	2520 (22.9%)	318 (26.8%)	1579 (26.8%)
2014	426 (19.4%)	2130 (19.4%)	311 (26.2%)	1543 (26.2%)
2015	458 (20.9%)	2290 (20.8%)	252 (21.3%)	1250 (21.2%)
2016	432 (19.7%)	2160 (19.7%)	165 (13.9%)	824 (14.0%)
2017	376 (17.1%)	1880 (17.1%)	103 (8.7%)	509 (8.7%)
2018	--	--	37 (3.1%)	185 (3.1%)

**Table 2:** Associations between tertiles of TREC levels and risk of acute lymphoblastic leukemia and acute myeloid leukemia.

	California Odds Ratio (95% CI)	Texas Odds Ratio (95% CI)
Acute lymphoblastic leukemia		
Tertile 1	1.00 (reference)	1.00 (reference)
Tertile 2	0.60 (0.47, 0.75)	1.45 (1.05, 1.99)
Tertile 3	0.85 (0.68, 1.07)	1.49 (1.06, 2.08)
Acute myeloid leukemia		
Tertile 1	1.00 (reference)	1.00 (reference)
Tertile 2	0.38 (0.22, 0.66)	0.96 (0.54, 1.72)
Tertile 3	0.47 (0.27, 0.82)	0.68 (0.36, 1.31)

Abbreviations: 95% CI, 95% confidence interval.

## Data Availability

The data is available through formal application to the Texas and California Newborn Registries and Cancer Registries.

## Abbreviations

The following abbreviations are used in this manuscript:

95% CI	95% confidence interval
ALL	acute lymphoblastic leukemia
AML	acute myeloid leukemia
CNS	central nervous system
IBD	intrahepatic bile duct
PCR	polymerase chain reaction
SCID	severe combined immunodeficiency
TREC	T-cell receptor excision circle
